# Development of diverse coding metamaterial structure for radar cross section reduction applications

**DOI:** 10.1038/s41598-022-14911-6

**Published:** 2022-06-29

**Authors:** Tayaallen Ramachandran, Mohammad Rashed Iqbal Faruque, Mohammad Tariqul Islam, Mayeen Uddin Khandaker, K. S. Al-mugren

**Affiliations:** 1 Space Science Center (ANGKASA), Universiti Kebangsaan Malaysia, UKM, 43600 Bangi, Selangor Malaysia; 2 Department of Electrical, Electronic & Systems Engineering, Universiti Kebangsaan Malaysia, UKM, 43600 Bangi, Selangor Malaysia; 3 Centre for Applied Physics and Radiation Technologies, School of Engineering and Technology, Sunway University, 47500 Bandar Sunway, Selangor, Malaysia; 4 Department of Physics, College of Science, Princess Nourah bint Abdulrahman University, 84428, Riyadh, 11671 Saudi Arabia

**Keywords:** Electrical and electronic engineering, Characterization and analytical techniques

## Abstract

Despite their widespread use for performing advanced electromagnetic properties, metamaterial suffers from several restrictions in this technological era. Generally, technology affects the way individuals communicate, learn, think and plays an important role in society today. For this reason, there has been a surge of interest in a coding metamaterial field that possesses the ability to manipulate electromagnetic waves and realize different functionalities. This research work investigates circular-shaped coding metamaterial for microwave frequency applications through several analyses. First, the 1-bit coding metamaterial that is made up of only “0” and “1” elements with 0 and π phase responses by adopting two types of unit cells such as square-shaped Rogers RT6002 substrate material with and without metamaterial structure were analysed in this work. The proposed element ‘1’ successfully manifests several more than 180○ phase responses at several frequency ranges, for instance, 7.35 to 9.48 GHz, 12.87 to 14.25 GHz and 17.49 to 18 GHz (C, X, and Ku-bands), respectively. Besides that, three types of coding sequences were proposed and the radar cross-section (RCS) reduction values of the designs were numerically calculated by utilising Computer Simulation Technology (CST) software. Meanwhile, the single-layered coding metamaterial with 6 lattices was compared with double and triple-layered metamaterial structures. At 2 GHz, the triple-layered structure exhibit reduced RCS values with near to − 30 dBm^2^ for all coding sequences. Therefore, the transmission coefficient results of the triple-layered coding metamaterial sequences were numerically calculated. Several advanced coding metamaterial designs were constructed and the properties were discussed in terms of RCS values and scattering patterns. Meanwhile, the scattering and effective medium parameters of the unit cell metamaterial structure were also analysed in this work. In a nutshell, the 1-bit coding metamaterial in a controlled sequence can control electromagnetic waves and realize different functionalities.

## Introduction

Research investigations regarding metamaterial have become familiar among the scientific community in the past few decades. Potential applications of metamaterial are diverse and include electromagnetic absorption reduction, sensor, terahertz frequency application, metamaterial filter, metamaterial absorption, etc. are successfully adapted metamaterial design structures to gain unique results^1–5^. Generally, the metamaterial design structure has its unique way of light interacting when compared to conventional materials. Conventional material can be easier found in nature but lacks in producing extraordinary electromagnetic properties. For instance, left-handed behaviour is very possible in man-made design structures instead of conventional materials. However, the current advanced technology is required beyond normal metamaterial structure design to optimise the performance to utilise in many application fields. Moreover, for specific applications likely metamaterial antenna, the conventional material is difficult to manufacture in large quantities. Meanwhile, the metamaterial absorption commonly has a very low narrow absorption band and shows a dispersion phenomenon outstandingly in the far-infrared domain. Therefore, many new concepts and ideas are developed in recent years such as metasurface, mechanical metamaterial, photonic metamaterial, acoustic metamaterial, plasmonic metamaterial, etc.

On the other hand, the coding metamaterial is the next step in advanced technology due to its properties to manipulate electromagnetic (EM) waves and realise different functionalities. Besides that, limited coding metamaterial studies are carried out up to date but have a great potential to be applied in many applications. However, research investigations to reduce RCS values were carried out by adopting many approaches. Invisibility cloaking is one of the methods used in RCS reduction where the EM waves are forced to bend around the target^6,7^. Meanwhile, a perfect absorber by adopting metamaterial is another approach to reducing RCS value by absorbing all the incident EM waves^8,9^. However, in this work, we adopted a newly discovered mechanism for reducing the RCS value by redirecting EM waves to all directions utilising a 1-bit coding metamaterial. This coding pattern was realised by adopting special “0” and “1” elements which compose of two types of material. Although limited studies were carried out in this field, Cui et al.^10^ successfully proposed digital metamaterial through two steps. The author successfully produced coding metamaterial by utilising two types of unit cells to mimic “0” and “1” elements. 1-bit and 2-bit coding analyses with various coding sequences were investigated and analysed in this research work. The work^11^ proposed a concept of frequency coding metamaterial to manifest various controls of EM energy radiations. This analysis utilised a fixed spatial coding sequence when changes in frequency occurred. Besides that, the author introduced a digitalized frequency sensitivity where the units were encrypted with “0” and “1” digits to realise low and high phase sensitivity. This sensitivity is used to describe the coding property of the unit cell. Meanwhile, in 2020, Cuong et al.^12^ investigated a broadband coding metamaterial for microwave absorber applications. The proposed work was designed, simulated and measured by carrying out various optimisation parametric studies. The initial unit cell metamaterial design with four types of coding blocks such as 2 × 2, 3 × 3, 4 × 4, and 6 × 6 were analysed and used for the construction of numerous 12 × 12 topologies with a sensible dimension. On the other hand, Liu^13^ introduced a controllable random metasurface which was possible by adding only a simple random coding sequence to the gradient coding sequence. This work aims to influence the probability of random scattering exhibited in a specific range of angles. On the other hand, the coding metamaterial has also been utilised in the terahertz frequency application recently. Jin et al.^14^ proposed a 1-bit flexible coding metamaterial with “0” and “1” elements by adopting two types of unit cells to shape the reflection and scattering of terahertz waves. In this research work, the analysis of low-reflection and scattering metamaterial in a wide terahertz range was performed. In 2021, Yang et al.^15^ investigate the tailoring of the scattering properties of a coding metamaterial by utilising machine learning. This work demonstrated two different reflection phase unit cells and the built-up model of semi-analytical for the unit cells. To optimise the coding matrix, the genetic algorithm is coupled with the scattering pattern analysis.

A systematic review of a prospective observational study by Zhang et al.^16^ presents recent progress of three newly discovered metamaterials known as coding, digital and programmable metamaterials. In this work, a discussion on the ability to control the EM waves in real-time and the building of multi-purpose devices was analysed. Both the 1-bit and 2-bit coding metamaterial concepts have also been investigated and analysed in this work. The investigation of optimised coding diffusion metasurface to reduce RCS values was performed by Ali et al.^17^ in 2019. This work also adopted a 1-bit coding metamaterial with two types of unit cells to mimic “0” and “1” elements. The full dimension of the proposed coding metamaterial is 264 × 264 × 3 mm^3^. Meanwhile, Liu et al.^18^ introduced a concept of anisotropic coding metamaterial for terahertz frequency applications. Therefore, the different directions of coding behaviour are dependent on the polarization status of terahertz waves. The proposed tailored coding elements on the ultrathin and flexible polarization-controlled anisotropic metasurfaces were also experimentally investigated.

On the other hand, several unique RCS reduction analyses were carried out, for instance, Mirkovic et al.^19^ and Zhou et al.^20^ in 2016 and 2021, respectively. Mirkovic analysed for the first time the electromagnetic modelling techniques (typically applied to man-made objects) can accurately predict organismal radio scattering characteristics from an anatomical model by adopting the method of moments implemented in the WIPL-D software package. Meanwhile, Zhou proposed a comprehensive design method based on the sorting factor Pareto solution to reduce the RCS value. On the other hand, many metamaterial and metasurface designs were investigated for the C, X, and Ku-bands in recent years. In 2019, Barde et al.^21^ investigated a very compact simple double square-shaped design of a metamaterial absorber for Ku and K band applications. Meanwhile, the similar first author proposed a wideband metamaterial absorber for Ku band application in 2020^22^. Furthermore, Roy et al.^23^ proposed an ultra-thin wideband metasurface polarization converter that is utilised for linear conversion i.e., X polarized to Y polarized and vice versa. All these three research works adopted ANSYS HFSS 19.1 software to perform all the numerical simulations.

According to the comprehensive survey, the previous studies reveal that many coding metamaterials were applied in a wide range of applications. However, this new coding metamaterial field required further research investigation to optimise the outcomes in the desired applications. Besides that, few of the previous studies adopted a bigger scale of coding metamaterial structure. Electronic devices with the miniaturisation concept have become the latest technological growth in industrial applications. Therefore, this research investigates a small-sized circular-shaped coding metamaterial structure by adopting a 1-bit coding sequence. Moreover, the introduced coding metamaterial effectively reduced RCS values in the frequency range from 2 to 18 GHz. Besides that, the multi-layered metamaterial structure by adopting the coding concept has been analysed in this work.

## Coding metamaterial

**Figure 1:**
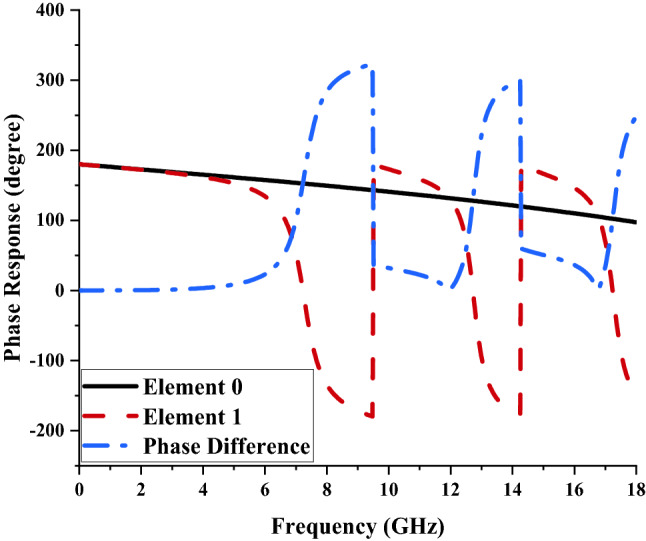
Phase response of elements ‘0’ and ‘1’.

All the analysis data in this research investigation are based on coding metamaterial concepts. Therefore, 1-bit coding metamaterial was adapted and initially unique metamaterial unit cells composed of binary digital elements such as “0” and “1” with 0 and 180^○^ phase responses were considered. These elements in a controlled coding sequence can realize different functionalities and manage to manipulate the EM waves. Figure 1 illustrates the phase response properties of both proposed elements such as ‘0’ and ‘1’ with the phase difference curve. The proposed element ‘1’ successfully manifests several more than 180^○^ phase responses at several frequency ranges, for instance, 7.35 to 9.48 GHz, 12.87 to 14.25 GHz and 17.49 to 18 GHz (C, X, and Ku-bands), respectively. Both of the unit cells utilised similar substrate material and dimensions (further details in “Element “1” metamaterial design”). The element “0” was constructed by adapting only Rogers RT6002 as substrate material while element “1” has an additional circular-shaped metamaterial structure. Although the stated elements can be simply defined as perfectly electric and magnetic conductors, reaching a higher frequency band requires a metamaterial structure designed on the substrate material to achieve binary elements^10^. The pre-existing conventional metamaterial act as analog technology which manipulates the EM fields by utilising effective medium parameters. However, the various coding sequences consisting of “0” and “1” elements can easily control the EM fields.

### Radar cross-section

**Figure 2:**
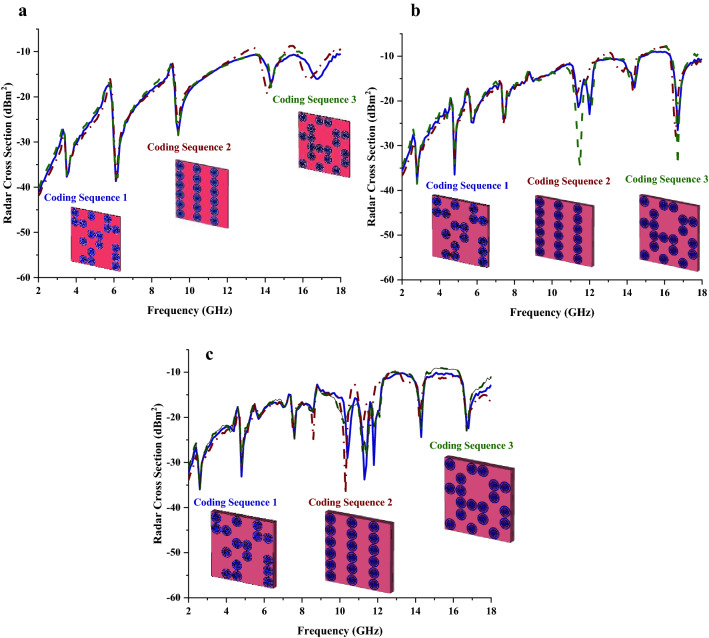
RCS results of three different metamaterial coding sequences for; (**a**) Single-layer, (**b**) Double-layer, (**c**) Triple-layer.

Several coding sequences by utilising the trial and error method such as those graphically portrayed in Fig. 2a–c were adapted and the RCS values were compared by the number of metamaterial layers in each subfigure. The three types of coding sequences have the arrangement of unit cells as demonstrated in Table 1. Therefore, using the trial and error method, these sequences were arranged in different forms to analyse the performances changes. Although this simple trial and error is typically used to discover new ideas, it also plays an important role in the scientific method as well. The optimization of the coding sequence by utilising “0” and “1” elements with 0 and π phase responses can achieve the best RCS reduction values. Radar cross-section is typically used to measure how detectable an object is by radar. The target that is located from the radar at a distance of R can greatly affect the definition of RCS and can be expressed as in Eq. (1).

1 $$\sigma = 4\pi {\text{R}}^{2} \cdot \frac{{P_{r} }}{{P_{i} }}$$

**Table 1:** Arrangement of both elements in each proposed coding sequence.

Coding sequence	Row 1	Row 2	Row 3	Row 4	Row 5	Row 6
1	011001	001001	010101	001100	110,011	101,010
2	101,010	101,010	101,010	101,010	101,010	101,010
3	101,010	010101	011101	110,010	010011	101,100

σ = Radar cross-section [m^2^ or dBm^2^]; R = distance between the target and radar receiver [m]; P_r_ = Backscattered or reflected power density [Watt/m^2^]; P_i_ = incident power density on target [Watt/m^2^].

Besides that, the RCS is defined as regards square meters or in the more conventional unit which is known as dBsm (dBm^2^). In other words, the RCS is also known as the ratio of the backscattered power density (P_r_) to the incident power density (P_i_) on the target. A few key factors influence the RCS values, for example, the signal frequency, the physical geometry of material, the direction of radar and the polarization of the scattered signal. There are two types of RCS values that can be measured such as monostatic and bistatic. The receiver and transmitter antennas are located at the same place for monostatic RCS while the bistatic RCS have different antenna positions. Generally, different structural composite materials will manifest dependent RCS values in any frequency range.

Therefore, one of the objectives of this research work is to investigate coding metamaterial on a smaller scale known as the miniaturization concept to realise an optimised RCS reduction value. The 6 lattices were adopted in this research work and all the optimised codes for the analysis are based on 6 × 6 with “0” or “1” elements. The monostatic RCS simulation results of the proposed coding metamaterial were obtained by utilising the commercially available software CST Microwave Studio. It is visible that, all the coding sequences manifest almost similar optimised RCS reduction values as shown in Fig. 2a which have near to − 10 dBm^2^ at the frequency ranges of 14 to 18 GHz. Note that, the RCS values at 2 GHz were slightly moved from − 40 dBm^2^ to − 30 dBm^2^ when metamaterial layers were increased. Besides that, the RCS reduction curves pattern also have minor discrepancies for double layer coding metamaterial structure. For example, at the fifth RCS reduction curve, coding sequence 3 exhibits higher magnitude values when compared to the other two sequences. Meanwhile, coding sequence 1 has double curve points with magnitude values less than − 30 dBm^2^. On the other hand, the triple-layer coding metamaterial sequences have better outcomes compared to the other two layers.

**Figure 3:**
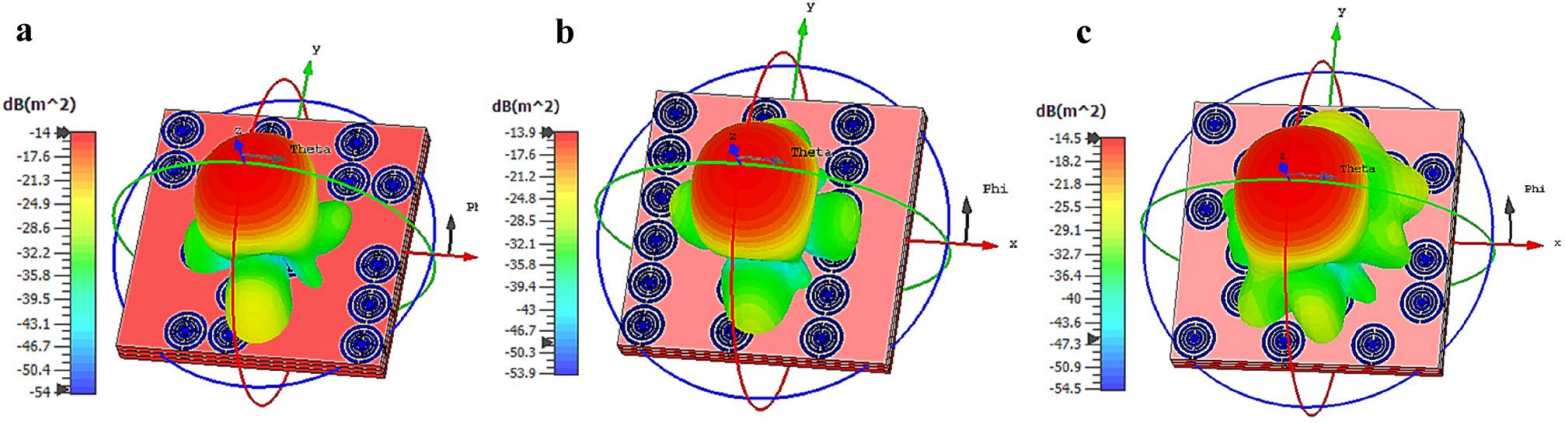
The full-wave simulation results of triple layer coding metamaterial for: (**a**) Coding sequence 1, (**b**) Coding sequence 2, (**c**) Coding sequence 3.

The three-dimensional scattering patterns of the multi-layered coding metamaterial are presented in Fig. 3a–c. This figure helps to observe the bistatic scattering features of the proposed three types of coding sequences. The incident waves scattered in an almost similar pattern for all three multi-layered designs. However, a slight difference can be visible where the Coding Sequence 1 has the least scatter beams and the number of beams increases as the changes in the sequences were made. Moreover, Coding Sequence 2 has the lowest bistatic RCS values at − 13.9 dBm^2^ and the comparison revealed only less than 5% of discrepancies occurred. On the other hand, Table 2 demonstrates the comparison of several RCS reduction application research and proposed design. Overall, the reference^24^ manifests the highest RCS reduction values but it has the larger metamaterial compared to the rest of the designs. Meanwhile, the reference^25^ possesses the second smallest metamaterial design but it only exhibits the least reduction value. Moreover, the reference^10^ successfully reduced the RCS values to − 23 dBm^2^ by adopting a 100 × 100 mm^2^ metamaterial design. However, the advanced technology requires miniaturised design structures to be applied in a wide range of applications. Therefore, a novel 48 × 48 mm^2^ coding metamaterial design was proposed to gain optimised RCS reduction applications.

**Table 2:** Several advanced coding metamaterial structures and RCS values.

References	Size (mm^2^)	Frequency range (GHz)	Design	RCS reduction (dBm^2^)
^10^	100 × 100	7 to 14	Coding metamaterial	− 23
^24^	212.5 × 95	5 to 8	Metamaterial	6
^25^	80 × 80	2 to 18	Metamaterial	− 30.1
Proposed	48 × 48	0 to 18	Coding metamaterial	Near − 10

### Scattering parameters of coding metamaterial

**Figure 4:**
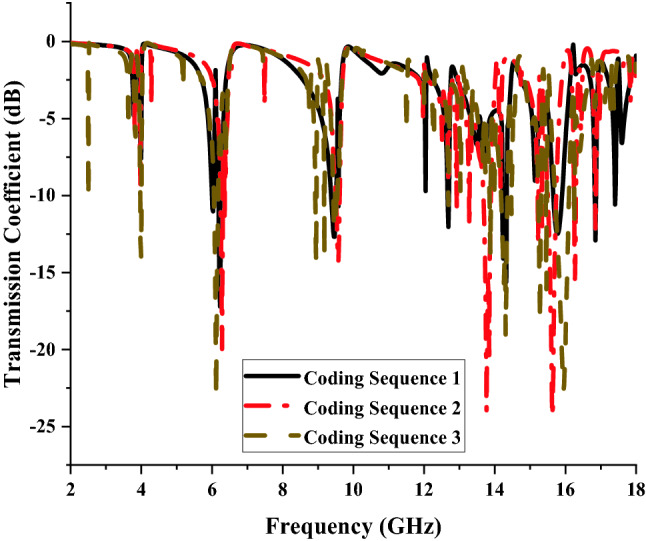
Transmission coefficient of coding metamaterial for single layers.

Scattering parameters also known as S-parameters define the electric behaviour of linear electrical networks on metamaterial when experiencing electrical signals in various steady-state stimuli. Due to the triple-layer coding metamaterial produces better RCS reduction values, therefore transmission coefficient results of these design structures were analysed in this paper. Each coding sequences manifest various transmission coefficient results. The comparison of these three sequences reveals that two to five resonance frequencies were manifested below − 15 dB (as shown in Fig. 4) and the outcomes are influenced by the coding sequence arrangement of “0” and “1” elements. We used various combinations of coding sequences where the element “1” was increased in the following sequences. For example, the total number of element “1” used in Coding Sequence 1 was 17 and for the following sequence was increased by 18. The changes in the coding patterns clearly show an increment in the number of resonance frequencies. The Coding Sequence 3 was able to manifest five peak points while sequences 1 and 2 only produce two and four resonances, respectively.

### Advanced coding metamaterial design

The second objective of this research paper is to analyse multiple advanced coding metamaterial designs by adapting Coding Sequence 1 arrangement with similar proposed elements. Several cuboid designs that possess single or multi-layered structures were constructed in this analysis as illustrated in Table 3. Designs 1 and 2 were constructed with single and triple-layered Coding Sequence 1 arrangement respectively. Therefore, Designs 1 and 2 exhibit RCS values less than 0 (on the positive axis), while the rest of the design structures were able to reach near − 10 dBm^2^. Although the first or last two designs exhibit almost similar RCS values, the design produced diverse scattering patterns. Design 1 has a sharp and slightly higher scattered beams pattern when compared to other designs. Meanwhile, these bistatic scattering features indicated that Design 2 has the least value by reducing the RCS value to 4.48 dBm^2^. Therefore, it indicates that various coding metamaterial sequences with diverse shapes will influence the RCS and scattering patterns. In a nutshell, the RCS values were successfully reduced by adopting advanced coding metamaterial design and it will the novelty of this research work.

**Table 3:**
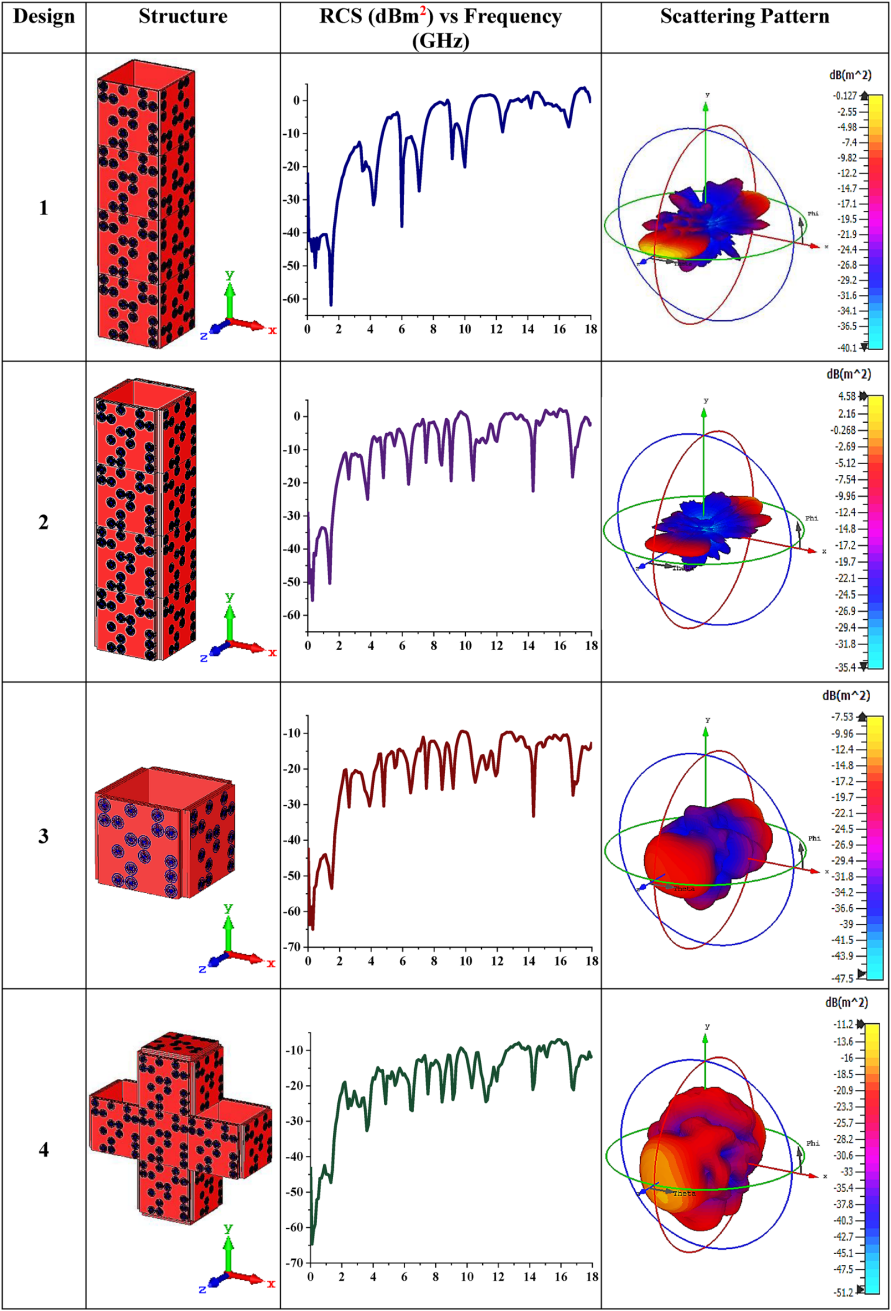
Several advanced coding metamaterial structures and RCS values.

### Validation

**Figure 5:**
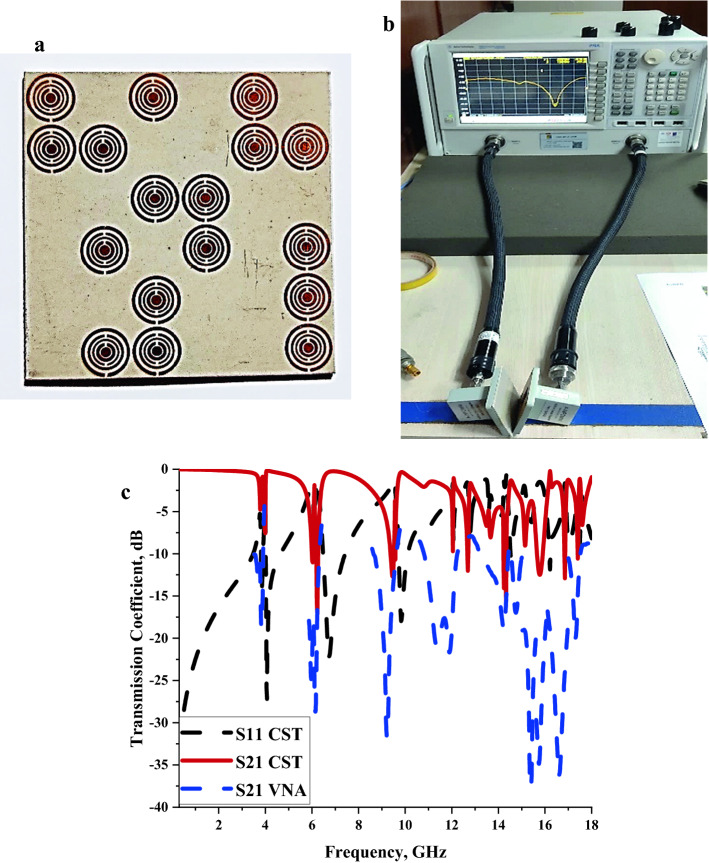
The S21 outcome validation of single-layered Coding Sequence 1; (**a**) fabricated coding metamaterial, (**b**) VNA setup, (**c**) transmission coefficient.

In this subsection, the S21 result of the single-layer Coding Sequence 1 metamaterial design was validated by performing a measurement process in Agilent N5227A Vector Network Analyser (VNA). Five types of adapters were used in this experiment, namely A-INFOMW WG to Coaxial adapter P/N: 187WCAS, A-INFOMW WG to adapter P/N: 137WCAS, A-INFOMW WG to adapter P/N: 112WCAS, A-INFOMW WG to adapter P/N:75WCAS and A-INFOMW WG to adapter P/N:51WCAS. Each waveguide port has different working frequency ranges. Therefore, the fabricated metamaterial was placed in between these waveguide ports respectively, to measure the S21 responses to Fig. 5a–c illustrate the fabricated coding metamaterial, VNA setup and the S21 results. As demonstrated in Fig. 5c, the comparison of numerically simulated and measured data reveals promising outcomes. Although all the resonances frequencies exhibit almost similar peak points, the magnitude values significantly differ from each other. This phenomenon may be caused by calibration error that has a great influence on the data produced. Besides that, the proposed circular split rings are small in size, thus unintentional flaws might have happened when removing excess copper from the substrate material. The numerically simulated S21 results possess only two peal points that exhibit more than − 15 dB magnitude values, for instance, at 6.23 and 14.33 GHz. However, all the measurement results exhibit magnitude values more than the − 15 dB and reach the maximum at 15.38 GHz with a − 37.78 dB magnitude value.

### Element “1” metamaterial design

The unit cell design that assigns element “1” was analysed in detail of its physical properties and characteristics based on the simulation and measurement data. This design was constructed by satisfying the main constraint variable which is the miniaturization concept.

#### Materials and methods

Only two types of materials were utilised in this research investigation. Firstly, Rogers RT6002 dielectric substrate material with 1.524 mm (r_t_) thickness was adopted. This material possesses a dielectric constant of 2.94 and tangent loss of 0.012. Meanwhile, a material with a thickness (c_t_) of 0.035 mm and conductivity (σ) of 5.80 × 10^7^ S/m known as copper was adopted to construct the circular-shaped metamaterial structure on the substrate material.

#### Metamaterial construction

**Figure 6:**
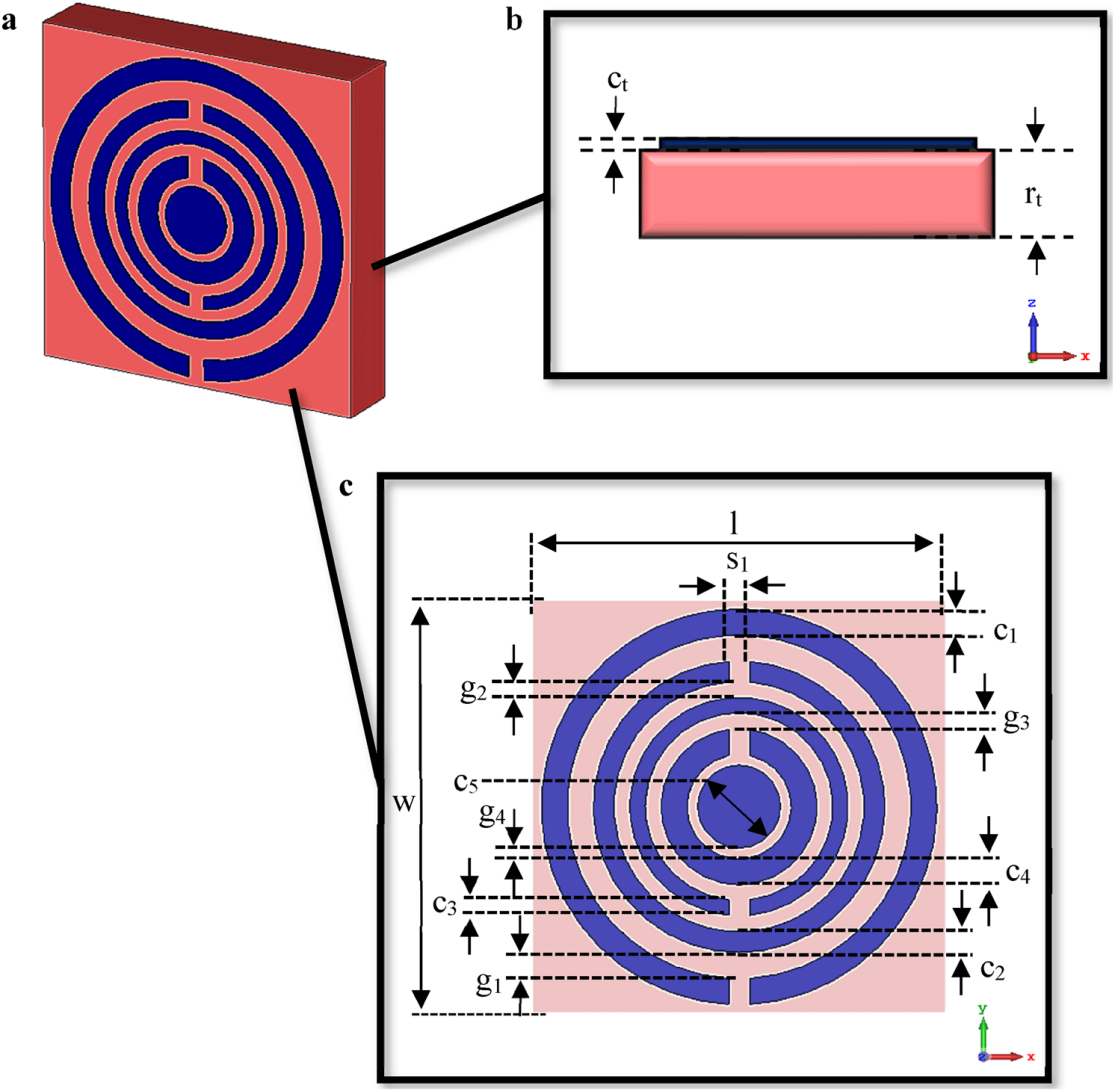
Graphical illustration exported from CST software: (**a**) Metamaterial design, (**b**) Side view, (**c**) Top view.

Figure 6 demonstrates the graphical illustration of the unit cell circular-shaped metamaterial exported from computer simulation software. The proposed “1” element metamaterial as shown in Fig. 6a has four circular rings and one circle at the centre of the design. Various ring thickness (c_n_) was utilised in this metamaterial design such as 0.50, 0.40, 0.30, and 0.50 mm from larger to smaller rings respectively. Meanwhile, the circle at the centre was designed with an outer diameter of 0.80 mm and the overall dimensions were selected by the method known as the trial and error approach. Although this straightforward approach was utilised, there were various numerical simulation analyses based on the thickness of rings, gaps between the rings, and optimised number of rings performed by utilising CST software. On the other hand, the circular rings were arranged as a split-ring resonator concept and the 0.40 mm split gaps were adopted for all the circular rings that were positioned either on top or bottom as illustrated in Fig. 6c. The split-ring resonator is generally common to metamaterial which is an artificially constructed structure. This structure can manifest desired magnetic response in many types of metamaterial until 200 THz. Strong magnetic coupling is created for an applied electromagnetic field in these media and is not found in natural material. Besides that, negative permeability behaviour is exhibited by adopting a periodic array of split-ring resonators. A split-ring resonator has a split at one end and enclosed loops on the other side. This loop is made of nonmagnetic metal like copper and can be circular or square-shaped structures with required gaps. The penetration of magnetic flux in the metal rings will induce current rotation within and manifest their flux to improve or resist the incident fields. This phenomenon depends on the resonant properties of the split-ring resonator. The rest of the dimension details were tabulated in Table 4 and graphically demonstrated in Fig. 6b,c.

**Table 4:** Description of proposed CRM design.

Descriptions	Dimension (mm)
c_1_	0.50
c_2_	0.40
c_3_	0.30
c_4_	0.50
c_5_	0.80
g_1_	0.50
g_2_	0.30
g_3_	0.30
g_4_	0.20
s_1_	0.40
RT6002 Length, l	8.00
RT6002 Width, w	8.00
Metamaterial Thickness, c_t_	0.035
RT6002 Thickness, r_t_	1.524

*c_*n*_ = circular ring thickness (*n* = 1, 2, 3, 4, 5); *g* = gap between rings; *s* = subtraction length.

#### Numerical simulation and measurement method

**Figure 7:**
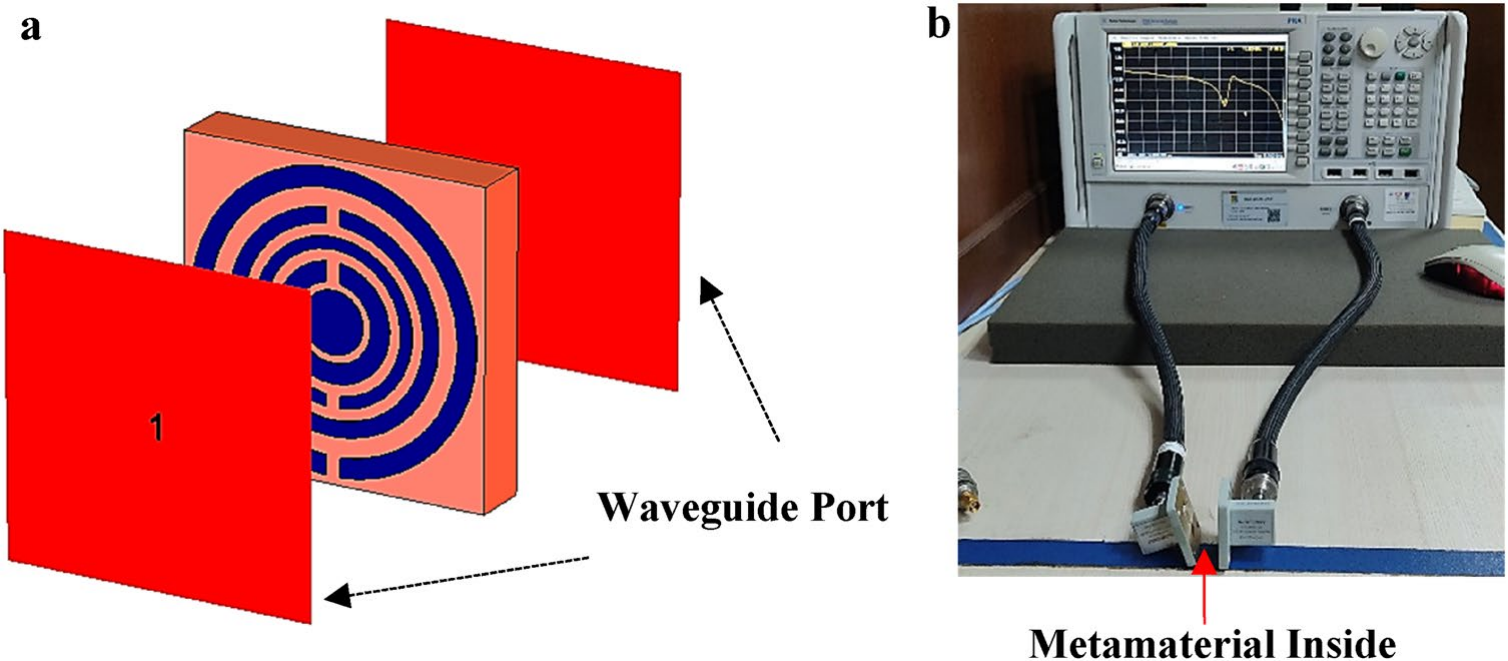
CST and experimental setup; (**a**) Unit cell metamaterial simulation; (**b**) VNA N5227A measurement setup.

All of the simulation analyses of the metamaterial transmission coefficient and RCS reduction of coding metamaterial were carried out by adopting well-known Computer Simulation Technology Microwave Studio (CST) software. The transmission coefficient of metamaterial numerical simulation was performed by adopting tetrahedral mesh (as illustrated in Fig. 7a) and frequency-domain solver while the RCS simulation utilised hexahedral mesh and time-domain solver. Meanwhile, the analysis of unit cell metamaterial utilised two waveguide ports positioned at the front and back of the structure. Besides that, these ports are paced in the z-axes direction with transverse electromagnetic mode. Therefore, the x and y-axis were set as perfect electric and perfect magnetic boundary conditions respectively. This research investigation focused on the frequency ranges from 2 to 18 GHz. On the other hand, the unit cell metamaterial analysis was performed by numerically simulating the structure to manifest the reflection (S11) and transmission (S21) coefficient results. These data were exploited to calculate the parameters of the metamaterial design, for instance, permittivity (ε), permeability (μ), and refractive index (n) values by utilising MATLAB software with the Robust method^26–28^. Equations from (2) to (5) express the formula to retrieve the z, n, ε, and μ parameters.

2 $$z = \pm \surd \frac{{\left( {1 + S_{11} } \right)^{2} - S_{21}^{2} }}{{\left( {1 - S_{11} } \right)^{2} - S_{21}^{2} }}$$

3 $$\begin{gathered} n = \frac{1}{{k_{0} d}}\left\{ {\left[ {In\left( {e^{{ink_{0} d}} } \right)} \right]^{\prime \prime } + 2m\pi - i\left[ {In\left( {e^{{ink_{0} d}} } \right)} \right]^{\prime } } \right\}, \hfill \\ e^{{ink_{0} d}} = \frac{{S_{21} }}{{1 - S_{11} \frac{z - 1}{{z + 1}}}} \hfill \\ \end{gathered}$$

4 $$\varepsilon = \frac{n}{z}$$

5 $$\mu = nz$$

The unit cell metamaterial defined as element “1” was further validated through experimental measurements. Usually, the board from an industrial manufacturing company is built with two types of materials which are defined as dielectric substrate layer and metal. This work adopted copper material as a metal for the metamaterial design construction and Roger RT6002 as substrate material. Hence, the excessive copper metamaterial will be removed based on the proposed metamaterial design during the fabrication process. The fabricated design was then measured through the VNA instrument (Fig. 7b illustrates the experimental setup). The unit cell was placed in between two waveguide ports that were connected to the VNA instrument by coaxial cables. Similar five types of adapters as for the measurement of coding metamaterial were adopted, namely A-INFOMW WG to Coaxial adapter P/N: 187 WCAS, A-INFOMW WG to adapter P/N: 137WCAS, A-INFOMW WG to adapter P/N: 112WCAS, A-INFOMW WG to adapter P/N:75WCAS and A-INFOMW WG to adapter P/N:51WCAS, respectively. An Agilent N4694-60001 device was utilised beforehand in the experiment process to secure precise readings without possible errors.

#### Scattering and effective medium parameters of unit cell

**Figure 8:**
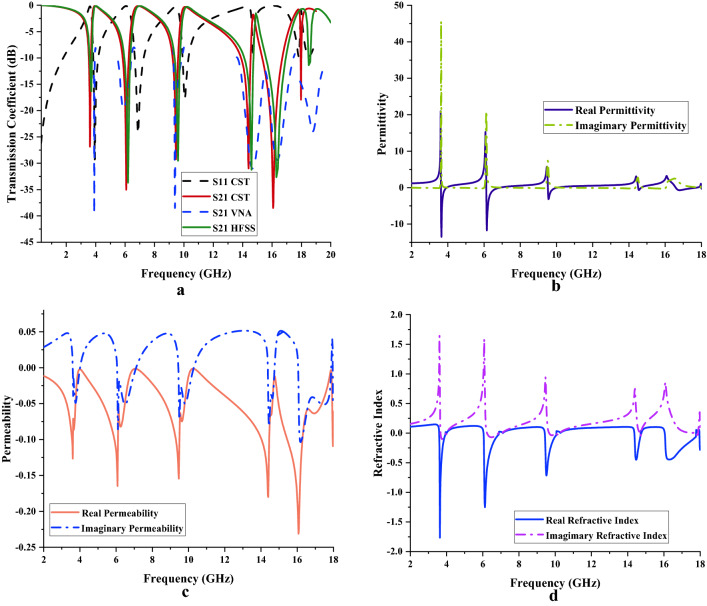
Scattering and effective medium parameters of unit cell metamaterial; (**a**) S11 and S21, (**b**) Permittivity, (**c**) Permeability; (**d**) Refractive Index.

Figure 8a–d exemplify the results retrieved from CST for the S11, S21, ε, μ, and n values. Based on Fig. 8a, quadruple bands were produced such as 3.61 (S-band), 6.08 (C-band), 9.46 (X-band), 14.40, 16.07 and 17.97 GHz (Ku-band) with acceptable magnitude values of − 26.86, − 35.03, − 29.25, − 31.01, − 38.50, and − 17.97 dB, respectively. The S21 measured data in Fig. 8a showed almost similar peak points except for the last resonance, such as 3.90, 5.89, 9.40, 14.66, 16.36, and 18.78 GHz, with acceptable magnitude values of − 39.55, − 19.71, − 38.49, − 31.00, − 29.40 and − 23.97 dB, respectively. Meanwhile, the S21 simulated results from the CST were further validated by adopting High-Frequency Structure Simulator (HFSS) software. In the first three resonance frequencies, both simulation results indicate identical peak points, while the rest start to show slight differences. The S21 responses in HFSS are as follows; 3.70, 6.20, 9.60, 14.60, 16.30 and 18.50 GHz with magnitude values of − 16.33, − 33.70, − 29.53, − 31.25, − 32.65 and − 11.39 dB, respectively.

The findings of permittivity values of unit cell metamaterial were demonstrated in Fig. 8b. Each resonance band manifests one negative behaviour on the adopted frequency range. However, the Ku-band possesses two negative behaviour curves with the lowest amplitude values when compared to others. The 3.63 to 3.93 GHz frequencies range manifest negative characteristics and acceptable amplitude values of − 9.12 to − 0.03 dB at the S-band. However, the negative permittivity in C-band starts at 6.14 GHz and lasted until 6.86 GHz. In this frequency range, the maximum negative ε value occurred at 3.65 GHz with an amplitude value of − 13.52 dB. Meanwhile, the frequency range between 9.54 and 10.03 GHz (X-band) showed negative ε behaviour with an optimised resonance frequency at 9.56 GHz with an amplitude value of − 3.15 dB. Finally, the introduced unit cell metamaterial depicted smaller two negative behaviour curves in Ku-band with maximum peak points at 14.55 GHz and 16.80 GHz with amplitude values of − 0.67 dB and − 0.70 dB, respectively. Moreover, the metamaterial design has negative permeability behaviour for whole the selected frequency range which is from 2 to 18 GHz as illustrated in Fig. 8c. The unit cell metamaterial manifests one peak value for each band except Ku-band. At the Ku-band the unit cell metamaterial exhibits triple peak values. Meanwhile, the maximum permeability value was recorded at 16.07 GHz with an amplitude value of − 0.23 dB. On the other hand, the refractive index (as shown in Fig. 8d) of metamaterial design also manifests a similar number of responses as permittivity behaviour. The maximum negative refractive index value was exhibited at 3.63 GHz with an amplitude value of − 1.77 dB (S-band).

#### Electric and magnetic field distribution of unit cell

**Figure 9:**
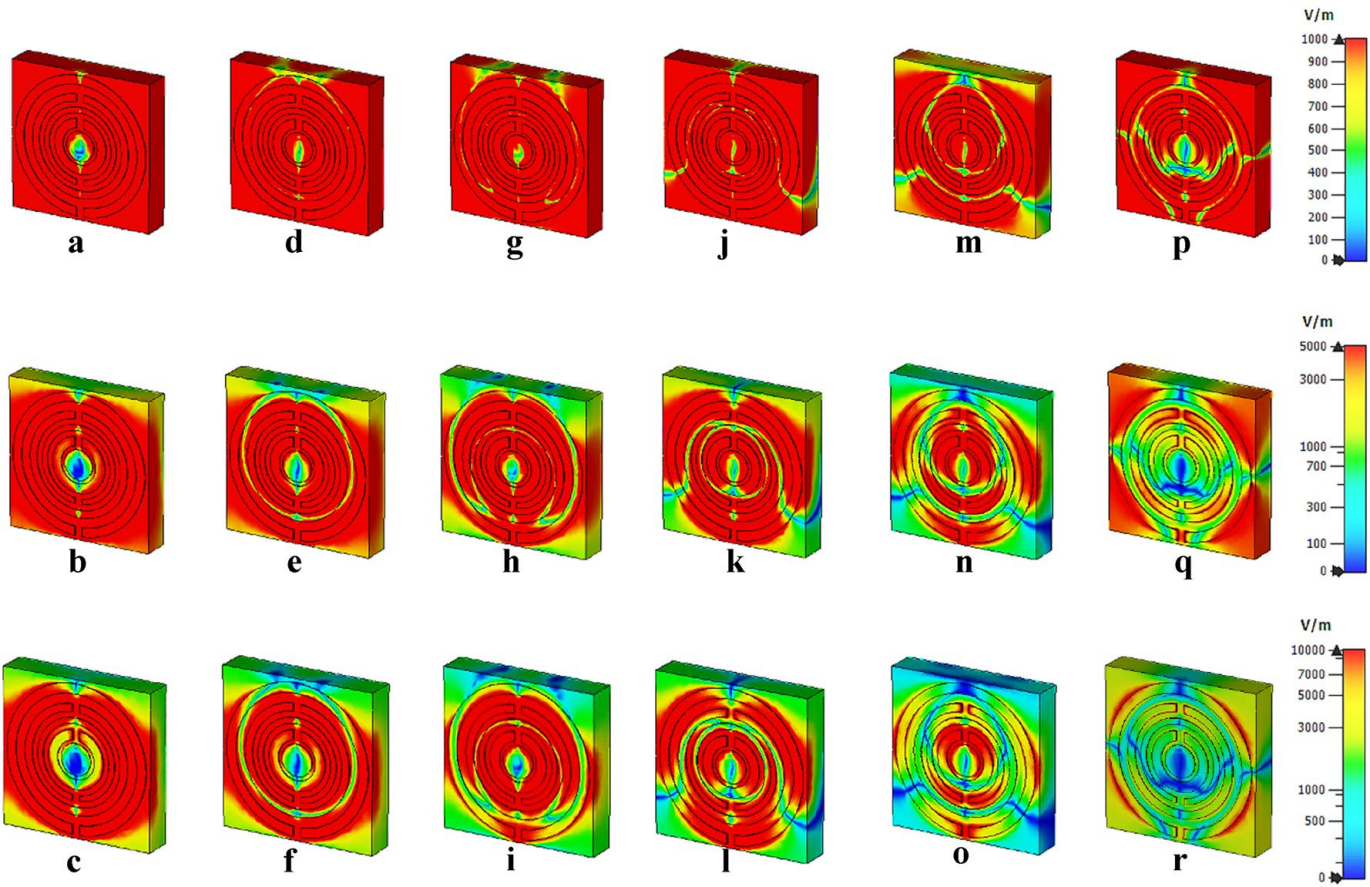
Electric field distribution with three limits at; (**a**–**c**) 3.61 GHz, (**d**–**f**) 6.08 GHz, (**g**–**i**) 9.46 GHz, (**j**–**l**) 14.40 GHz, (**m**–**o**) 16.07 GHz, (**p**–**r**) 17.97 GHz.

**Figure 10:**
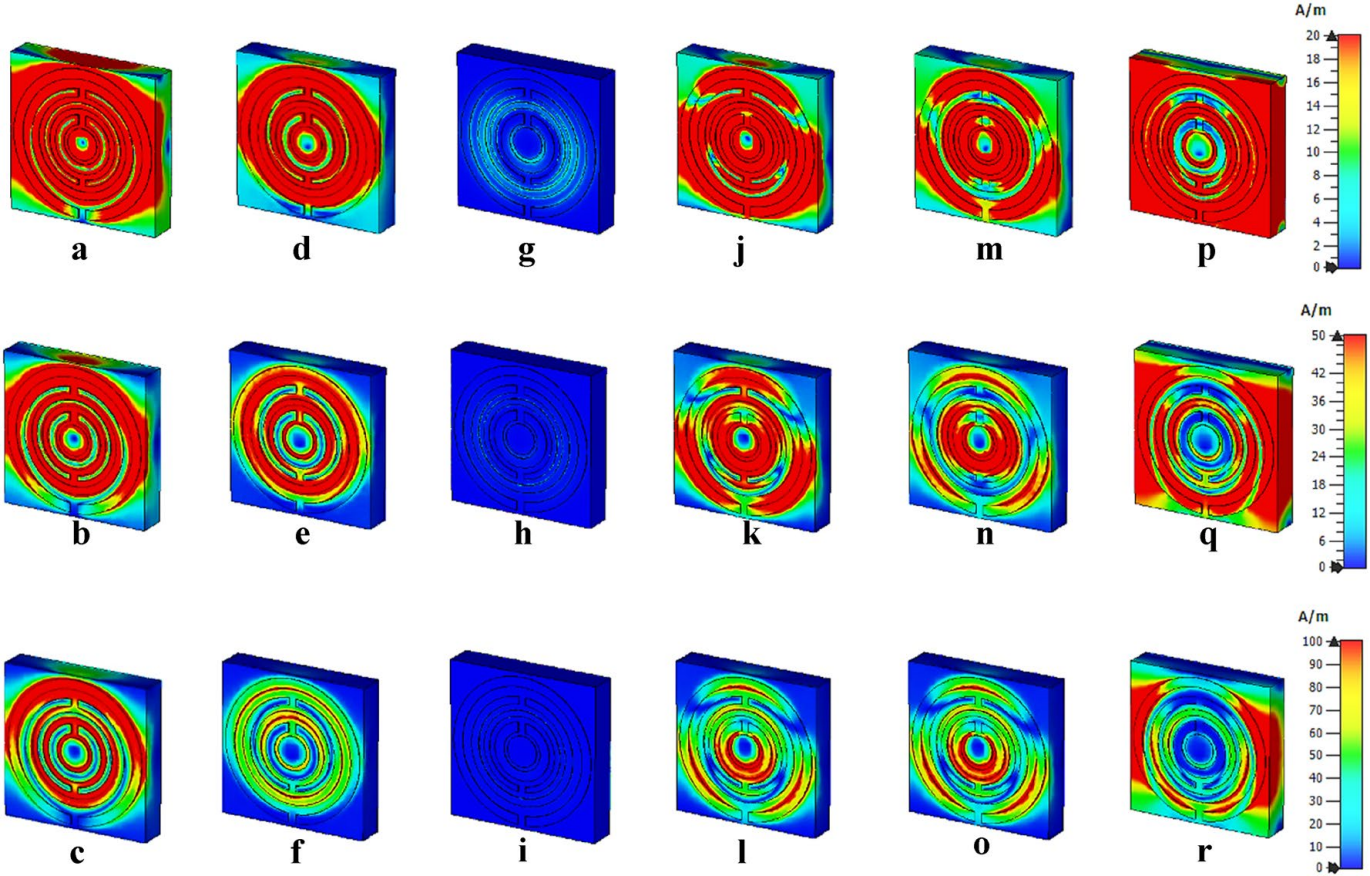
Magnetic field distribution with three limits at; (**a**–**c**) 3.61 GHz, (**d**–**f**) 6.08 GHz, (**g**–**i**) 9.46 GHz, (**j**–**l**) 14.40 GHz, (**m**–**o**) 16.07 GHz, (**p**–**r**) 17.97 GHz.

Figures 9 and 10 indicate the electric and magnetic field distributions of the proposed unit cell metamaterial. Generally, the physical characteristics of the electromagnetic field control the responses from these field distributions of the proposed design. This incident occurred in space due to the electric charges with time-varying behaviour. The desired resonance frequencies and outstanding accomplishments of the proposed design are dependent on the lossy and dispersive characteristics of a material. Three electric field distributions at three limits, for instance, 1000, 5000 and 10,000 V/m were adapted in this simulation. The examination disclosed a strong electric field detection at 1000 V/m and the responses nearly occurred on the whole design for every resonance frequency. Nevertheless, the intensity of the electric field was gradually reduced when the field strength was increased. Meanwhile, the 10,000 V/m limits had the least detection of distribution for all four resonance bands compared to the other field strengths. Besides that, around 75% of the exposure level was reduced for the last resonance frequency and it only focused on the outside of the first circular ring. Furthermore, the introduced metamaterial structure produced inconsistent magnetic field distributions for the chosen limits of 20, 50, and 100 A/m (as shown in Fig. 10). The magnetic field is generated due to the current induction when the wave passes through the split rings. The distribution fields show an irregular pattern as it reduced and then increased when the frequency increases. Moreover, at 9.46 GHz, the metamaterial exhibits almost zero magnetic field exposure when compared to others. Whereby, the field distribution was gradually increased after this peak point and reaches maximum exposure at 20 A/m. However, a standard query emerged from these discoveries which linked with the detection of these distributions on the dielectric substrate material. On the other hand, the copper metamaterial structure which is classified as metal-composed material is typically induced electric or magnetic field due to the free-electron within their physical structure. However, the dielectric material does not possess this type of free electron, but still, the field distribution takes place on its surface. This is caused by the delocalisation of the electron oscillation that is presented in the metal and substrate materials.

## Conclusion

This paper concludes by arguing the RCS values of coding metamaterial that are computed by utilising CST microwave studio. The 1-bit coding metamaterial was analysed in this work by adopting two types of unit cells to mimic “0” and “1” elements with 0 and π phase responses. Although the single-layered coding metamaterial for all sequences manifests almost similar RCS reduction behaviour, multiple layered metamaterials manifest different outcomes. Besides that, the transmission coefficient results of the multi-layered coding metamaterial have also been analysed here. Moreover, several advanced coding metamaterial cuboid designs were introduced and the RCS and scattering pattern of each structure were examined. To validate the numerical simulation results, the single-layered Coding Sequence 1 and unit cell metamaterial designs were fabricated and measured by using the VNA instrument. Moreover, the scattering and effective medium properties of the unit cell design were briefly discussed in this work. Briefly, the reduction of RCS values was successful by adopting the advanced coding metamaterial cuboid design and it will be the novelty of this research investigation. The introduced coding metamaterial is unique for a wide range of applications, such as reducing scattering features of targets, manipulating antennas radiation beams and manifesting various extraordinary properties of metamaterial. In summary, the achieved results fulfil the objectives of this work where the proposed coding metamaterial reduced RCS values. The further extended research investigation of coding metamaterial can be a valuable asset in this technological era.
